# Blood neurofilament light chain in Parkinson disease and atypical parkinsonisms: A protocol for systematic review and meta-analysis

**DOI:** 10.1097/MD.0000000000021871

**Published:** 2020-10-02

**Authors:** HongZhou Wang, WanHua Wang, HaiCun Shi, LiJian Han, PingLei Pan

**Affiliations:** a Department of Neurology, Kunshan Hospital, Affiliated to Jiangsu University, Kunshan; b Department of Neurology; c Department of Central Laboratory, The Yancheng School of Clinical Medicine of Nanjing Medical University, Yancheng, PR China.

**Keywords:** blood, meta-analysis, neurofilament light chain, Parkinson disease, parkinsonism

## Abstract

**Background:**

Neurofilament light chain (NfL), an index of neuroaxonal injury, is a promising diagnostic and prognostic fluid biomarker with high translational value in many neurodegenerative disorders. Blood NfL measurement has been an exciting and active field of research in idiopathic Parkinson disease (PD) and atypical parkinsonisms. However, blood NfL levels in these parkinsonisms from existing literature were inconsistent. No comprehensive meta-analysis has ever been conducted.

**Methods:**

Three major biomedical electronic databases PubMed, Embase, and Web of Science were comprehensively searched from inception to July 10, 2020. This protocol will be prepared based on the guidelines recommended by the statement of Preferred Reporting Items for Systematic Review and Meta-Analysis Protocols (PRISMA-P). Original observational studies that measured blood (serum/plasma) NfL concentrations in patients with parkinsonisms (multiple system atrophy [MSA], progressive supranuclear palsy [PSP], corticobasal syndrome [CBS], and dementia with Lewy bodies [DLB]), and healthy controls (HCs) will be included. Quality assessment of the included studies will be performed using the Newcastle Ottawa Scale (NOS). Meta-analyses will be conducted using the STATA software version 13.0. The standardized mean differences as the measure of effect size and 95% confidence intervals were calculated for each comparison of blood NfL levels. Heterogeneity analysis, sensitivity analysis, publication bias, subgroup analysis, and meta-regression analysis will be carried out to test the robustness of the results.

**Results:**

The meta-analysis will obtain the effect sizes of blood NfL levels in the following comparisons: PD versus HC, MSA versus HC, PSP versus HC, CBS versus HC, DLB versus HC, MSA versus PD, PSP versus PD, CBS versus PD, and DLB versus PD.

**Conclusions:**

The present meta-analysis will provide the quantitative evidence of NfL levels in idiopathic PD and atypical parkinsonisms, hoping to facilitate differential diagnoses in clinical practice.

**Registration number:**

INPLASY202070091.

## 1 Introduction

Parkinsonism is a neurological syndrome characterized by bradykinesia, tremor, rigidity, and postural instability, referring to a group of neurodegenerative disorders with considerable overlap in symptoms but with heterogeneity in presentation and pathology.[^1^,^2^,^3^] The most common cause of parkinsonism is idiopathic Parkinson disease (PD) that presents typical asymmetrical motor symptoms with slow progression and marked and sustained response to dopaminergic treatment.[^4^,^5^] Unlike idiopathic PD, atypical parkinsonisms, including multiple system atrophy (MSA), progressive supranuclear palsy (PSP), corticobasal syndrome (CBS), and dementia with Lewy bodies (DLB), show additional clinical signs like gaze palsy, apraxia, ataxia, early cognitive decline, and dysautonomia.[^1^,^2^,^6^] These atypical parkinsonisms generally progress more rapidly with poorer prognosis and poorer response to dopaminergic therapy than idiopathic PD.[^1^,^2^,^7^] Correct diagnoses of parkinsonian disorders are important for patient counseling, prognostic assessment, and therapeutic implications, but also research purposes. However, their differential diagnoses on clinical grounds remain challenging particularly in early disease stages.[^2^,^5^,^8^] Sustained efforts have been made to develop reliable biomarkers to aid their accurate diagnoses.[^2^,^9^,^10^,^11^,^12^,^13^,^14^,^15^]

Neurofilament light chain (NfL) is among the most promising candidate biomarkers of neuroaxonal injury irrespective of the underlying cause and has been extensively investigated in neurodegenerative diseases.[^16^,^17^,^18^,^19^,^20^,^21^] Recent meta-analyses have shown that cerebrospinal fluid (CSF) NfL levels were increased significantly in PSP,^[16]^ MSA,^[16]^ and DLB.^[17]^ By contrast, CSF NfL levels were not increased in idiopathic PD.^[16]^ CSF levels of NfL have shown the potential value in the discrimination of idiopathic PD from atypical parkinsonisms.[^22^,^23^,^24^,^25^] CSF and blood NfL levels are highly correlated that is related to neurodegeneration.[^26^,^27^,^28^] Compared to CSF that needs to perform a lumbar puncture, blood (serum/plasma) is an easier and more rapidly accessible biospecimen with relatively limited restrictions in clinical practice. With the development of ultrasensitive assay technologies in recent years, a growing number of studies have moved to investigate blood (serum/plasma) NfL levels in idiopathic PD and atypical parkinsonisms. Atypical parkinsonisms commonly showed increased blood NfL levels.[^27^,^28^,^29^,^30^] However, the degree of elevation varied considerably between these disorders. Besides, blood NfL levels in idiopathic PD were inconsistent across studies.[^26^,^27^,^28^,^29^,^30^,^31^,^32^] A meta-analysis is needed to quantitatively pool individual small studies to improve the power to detect differences. Thus, we performed a meta-analysis of available studies that analyzed blood (serum/plasma) NfL levels in idiopathic PD and atypical parkinsonisms. We will compare differences of blood (serum/plasma) NfL levels between patients with idiopathic PD and healthy controls (HCs), between patients with atypical parkinsonism subtypes and HCs, and between patients with idiopathic PD and atypical parkinsonism subtypes.

## 2 Methods

### 2.1 Search strategies

Three major biomedical electronic databases PubMed, Embase, and Web of Science were comprehensively searched from inception to July 10, 2020. The following search terms were used: (neurofilament OR (neurofilament light chain) OR nfl) AND ((Parkinson disease) OR parkinsonism OR Parkinson∗ OR (atypical parkinsonian disorders) OR (multiple system atrophy) OR (progressive supranuclear palsy) OR (Steele-Richardson-Olszewski syndrome) OR (corticobasal syndrome) OR (corticobasal degeneration) OR (dementia with Lewy bodies) OR (Lewy body dementia)) AND (blood OR serum OR plasma). Additionally, references of eligible articles and relevant systematic reviews/meta-analyses will be hand-searched.

This protocol will be prepared based on the guidelines recommended by the statement of Preferred Reporting Items for Systematic Review and Meta-Analysis Protocols (PRISMA-P).^[33]^

### 2.2 Eligibility criteria

#### 2.2.1 Inclusion criteria

Studies will be included if they: were original observational studies (including case-control, cohort, and cross-sectional studies) in English; included patients with idiopathic PD and atypical parkinsonisms (MSA, PSP, DLB, and CBS) according to the established diagnostic criteria; measured blood (serum/plasma) NfL concentrations in patients with parkinsonisms and HCs; provided sufficient information for meta-analysis (number of participants and mean and standard deviation [SD] for blood NfL levels for each group). If some data were not eligible, they will be transformed from available data or will be obtained using graph-based data mining methods.

#### 2.2.2 Exclusion criteria

The following exclusion criteria will be applied: studies were in the form of reviews, letters, editorials, conference abstracts, animal research, case reports, and protocols; studies enrolled repetitive patient samples or were overlapped with another study with a larger sample size. In case of longitudinal studies, we will only extract baseline data for analysis.

**Figure 1:**
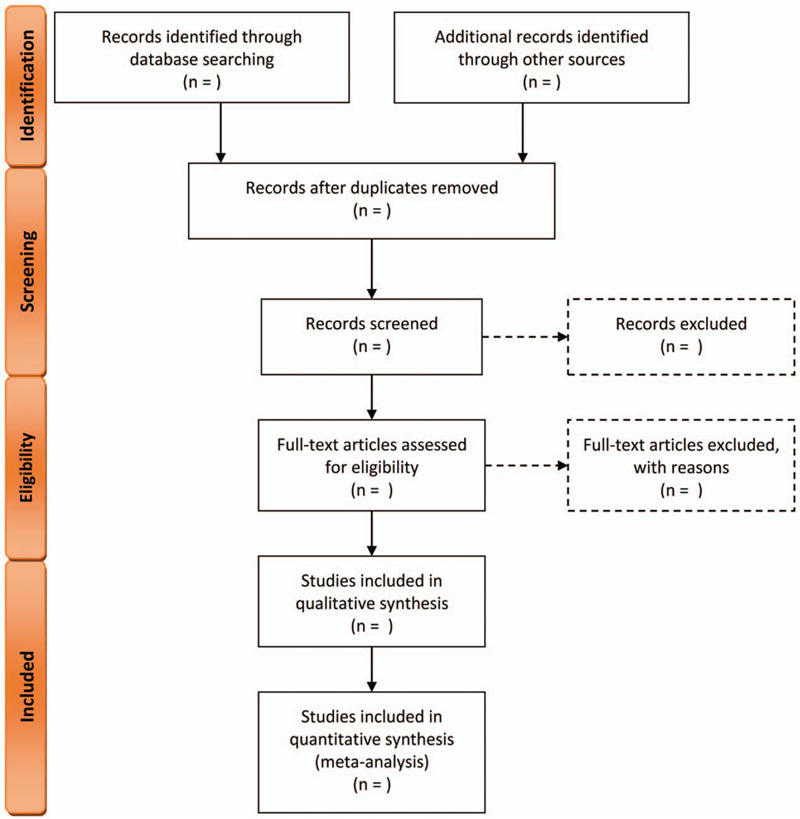
The flowchart of study selection following the PRISMA statement. PRISMA = Preferred Reporting Items for Systematic Review and Meta-Analysis.

The flowchart of study selection following the PRISMA statement^[34]^ is presented in Figure 1.

### 2.3 Data extraction

Data from eligible studies will be extracted using a standardized spreadsheet, including the following information: the first author's surname, year of publication, type of parkinsonisms, the numbers of patients and healthy controls, age, gender distribution, the mean and SD values of the blood NfL levels of each group, NfL analysis methods, analysis kit brand, disease duration, Hoehn and Yahr (H-Y) scale, Unified Parkinson's Disease Rating Scale, part III (UPDRS-III) score, and Mini-Mental State Examination (MMSE) score.

### 2.4 Quality assessment

Quality assessment of the included studies will be performed using the Newcastle Ottawa Scale (NOS).^[35]^ This scale includes 3 main fields of selection (0–4 scores), comparability (0–3 scores), and exposure (0–3 scores). The NOS score lower than 6 scores will be considered a low-quality methodology.

### 2.5 Data synthesis and statistics

Meta-analyses will be conducted using the STATA software version 13.0 (StataCorp, College Station, TX). The standardized mean differences (SMD) as the measure of effect size and 95% confidence intervals (CI) were calculated of each comparison of blood NfL levels (PD vs HC, MSA vs HC, PSP vs HC, CBS vs HC, DLB vs HC, MSA vs PD, PSP vs PD, CBS vs PD, and DLB vs PD). Study heterogeneity will be estimated with the I^2^ statistic. A fixed-effects model will be used if there was no obvious statistical heterogeneity with I^2^ less than 50%; otherwise, the random-effects model will be selected. I^2^ < 50%, between 50% and 75%, and I^2^ > 75% will be deemed as low, moderate, and high heterogeneity, respectively.

To examine whether overall results were influenced by a single study, sensitivity analyses will be performed. A funnel plot will be created and Egger test will be analyzed to test the potential publication bias. Subgroup analyses and meta-regression analyses will be conducted to examine whether potential moderators influence the meta-analysis effect sizes. Meta-regression analyses will be only performed when relevant data were available from more than 10 studies.

Study selection, quality assessment, and data extraction will be performed by one author and verified by another.

### 2.6 Ethics and dissemination

Ethics Committee approval was waived because this meta-analysis will be performed using the data from published studies that will not involve any human participants or animals. Once the meta-analyses are complete, it will be published in conferences or a peer-reviewed journal.

## 3 Discussion

NfL is a promising diagnostic and prognostic fluid biomarker with high translational value in many neurological disorders.[^36^,^37^,^38^] Due to its convenience and lesser invasiveness in clinical practice, blood NfL measurement has been an exciting and active field of research.[^36^,^39^] Idiopathic PD and atypical parkinsonisms are different in the clinical course, prognosis, and therapy needs. Recent studies have shown that blood NfL levels added to their differential diagnoses. [^27^,^40^] However, previous studies on NfL levels in idiopathic PD and atypical parkinsonisms were inconsistent. The meta-analysis will obtain the effect sizes of blood NfL levels in the following comparisons: PD versus HC, MSA versus HC, PSP versus HC, CBS versus HC, DLB versus HC, MSA versus PD, PSP versus PD, CBS versus PD, and DLB versus PD. The present meta-analysis will provide quantitative evidence of NfL levels in these disorders, hoping to facilitate differential diagnoses in clinical practice.

## Author contributions

**Conceptualization:** HongZhou Wang, LiJian Han, PingLei Pan

**Data curation:** HongZhou Wang, WanHua Wang

**Formal analysis:** HongZhou Wang

**Funding acquisition:** PingLei Pan

**Investigation:** HongZhou Wang, WanHua Wang, HaiCun Shi

**Methodology:** HongZhou Wang, WanHua Wang, HaiCun Shi

**Project administration:** LiJian Han, PingLei Pan

**Resources:** HongZhou Wang, WanHua Wang, HaiCun Shi

**Software:** HongZhou Wang, WanHua Wang

**Supervision:** LiJian Han

**Validation:** PingLei Pan

**Visualization:** HongZhou Wang, WanHua Wang

**Writing – original draft:** HongZhou Wang, WanHua Wang

**Writing – review & editing:** LiJian Han, PingLei Pan
